# Elimination of *Plasmodium falciparum* malaria in Tajikistan

**DOI:** 10.1186/s12936-017-1861-5

**Published:** 2017-05-30

**Authors:** Anatoly V. Kondrashin, Azizullo S. Sharipov, Dilshod S. Kadamov, Saifuddin S. Karimov, Elkhan Gasimov, Alla M. Baranova, Lola F. Morozova, Ekaterina V. Stepanova, Natalia A. Turbabina, Maria S. Maksimova, Evgeny N. Morozov

**Affiliations:** 1 Sechenov First Moscow State Medical University, Moscow, Russian Federation; 2 Republican Centre for Control of Tropical Diseases, Ministry of Health and Social Welfare, Dushanbe, Tajikistan; 3 World Health Organization, Regional Office for Europe, Copenhagen, Denmark; 4 Russian Medical Academy of Continuous Professional Education, Moscow, Russian Federation

**Keywords:** Malaria, Control, *P. falciparum* malaria, Elimination, Tajikistan, Anti-malaria measures

## Abstract

Malaria was eliminated in Tajikistan by the beginning of the 1960s. However, sporadic introduced cases of malaria occurred subsequently probably as a result of transmission from infected mosquito Anopheles flying over river the Punj from the border areas of Afghanistan. During the 1970s and 1980s local outbreaks of malaria were reported in the southern districts bordering Afghanistan. The malaria situation dramatically changed during the 1990s following armed conflict and civil unrest in the newly independent Tajikistan, which paralyzed health services including the malaria control activities and a large-scale malaria epidemic occurred with more than 400,000 malaria cases. The malaria epidemic was contained by 1999 as a result of considerable financial input from the Government and the international community. Although *Plasmodium falciparum* constituted only about 5% of total malaria cases, reduction of its incidence was slower than that of *Plasmodium vivax*. To prevent increase in *P. falciparum* malaria both in terms of incidence and territory, a *P. falciparum* elimination programme in the Republic was launched in 200, jointly supported by the Government and the Global Fund for control of AIDS, tuberculosis and malaria. The main activities included the use of pyrethroids for the IRS with determined periodicity, deployment of mosquito nets, impregnated with insecticides, use of larvivorous fishes as a biological larvicide, implementation of small-scale environmental management, and use of personal protection methods by population under malaria risk. The malaria surveillance system was strengthened by the use of ACD, PCD, RCD and selective use of mass blood surveys. All detected cases were timely epidemiologically investigated and treated based on the results of laboratory diagnosis. As a result, by 2009, *P. falciparum* malaria was eliminated from all of Tajikistan, one year ahead of the originally targeted date. Elimination of *P. falciparum* also contributed towards speedy reduction of *P. vivax* incidence in Tajikistan.

**Electronic supplementary material:**

The online version of this article (doi:10.1186/s12936-017-1861-5) contains supplementary material, which is available to authorized users.

## Background

Malaria was known in Tajikistan since ancient times. The first organized studies on malaria during the 1920s revealed that all the population of the mountainous valleys was affected by malaria. Anti-malaria activities on a large scale were organized during the 1930s when the number of registered cases was more than 176,000 [1]. The Malaria Programme in Tajikistan became a part of the National Malaria Eradication Programme of the USSR during the 1950s, and malaria was eliminated from the whole territory of the Republic by the beginning of the 1960s. Sporadically-introduced cases of malaria were reported thereafter through presumed transmission by infected mosquito Anopheles flying over the river Punj from border areas of Afghanistan.

The malaria situation deteriorated suddenly in 1991–1992 associated with armed conflict and civil unrest in the newly independent Tajikistan. The health system was paralyzed and malaria control activities ceased. The malaria situation deteriorated further in 1993 with the return of some 600,000 Tajik refugees from malaria endemic regions of Afghanistan and Pakistan. Following the influx of refugees, a large-scale malaria epidemic occurred in the following years with an estimated more than 400,000 malaria cases. *Plasmodium vivax* comprised the majority with *Plasmodium falciparum* constituting about 5% of total malaria cases. The civil war lasted until 1997. Containment of the malaria epidemic started in 1999 with considerable financial input from the Government and the international community. The details of this are described below.

## Geography of Tajikistan

**Fig. 1:**
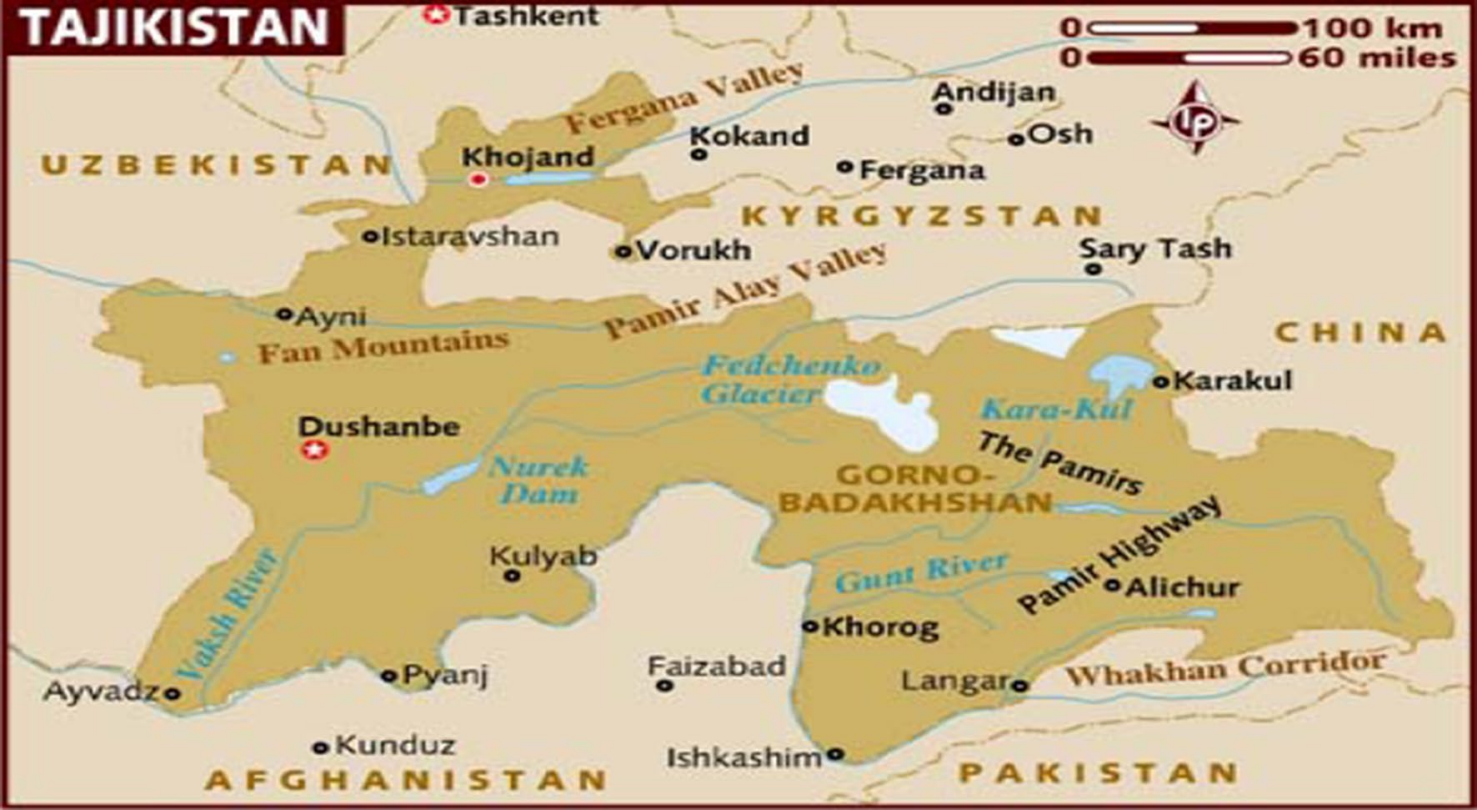
Map of Tajikistan

The Republic of Tajikistan is a mountainous state in Central Asia (Fig. 1).

There are more than one thousand glaciers and rivers in the Republic. Climate in Tajikistan is typically continental, dry, with considerable variations in temperature and rainfall pattern.

Total population of the Republic is 7,350,000 (2009) with density of population being 51.5 inhabitant/sq km. Dushanbe is the capital of the Republic and is the biggest city in the country, followed by Khojand, Kurgan-Tube and Kulyab. About 25% of total population resides in urban areas. Annual growth of population is 1.9 (2009), literacy rate—99.5%. Although only 10% of the country’s territory is suitable for agriculture, Tajikistan is basically an agricultural country. Rice and cotton are leading agricultural products [2].

## Health system of Tajikistan

The health system in Tajikistan is structured in accordance with the administrative and geographical division of the country. At the Republic level, there is a Ministry of Health and Social Welfare. At the provincial and Dushanbe city level, it is represented by the provincial health board and city executive board (*hukumat*). At the district and town levels, it is represented by hospitals and municipalities. The lowest level is the Primary Health Centre (*jamoat*). Primary health care services in urban and rural areas are provided by the Primary Health Centres, which offer diagnostic procedures, treatment of the most common illness and injuries, curative and preventive measures against parasitic and other diseases, immunization, community awareness raising and health education, mother-and-child health protection measures. Health centres play an essential role in the detection and follow up malaria cases. At each administrative level, there is a network of hospitals, health centres, laboratories and sanitary epidemiological services, which are responsible for communicable disease control including antimalarial interventions.

## History of malaria elimination in Tajikistan

### Elimination of malaria

Elimination of malaria in the Republic had been achieved by the National Malaria Programme by the beginning of the 1960s. *Plasmodium falciparum* was the first malaria species to be eliminated in the territory of the Republic by the end of the 1950s [1]. Residual *P. vivax* foci had persisted in some pockets of Gorno-Badakhshan Oblast (Western Pamir mountain system) and in the south of the Republic [3].

### Malaria situation in post-elimination period in Tajikistan (1963–1990)

Following malaria elimination, some sporadic cases of malaria were registered in the border areas with Afghanistan. The disease was thought to be associated with the introduction of infected vector(s) from Afghanistan. The capability of malaria vectors to fly across the narrow river bordering Punj district was proved experimentally [4].

The first indigenous cases were registered in 1971–1972 in areas still in close proximity to Punj River [5]. With the beginning of civil unrest in Afghanistan during the 1980s, the malaria incidence increased in the Tajikistan’s border districts as well (from 40 cases in 1980–460 indigenous cases in 1983). The trend of increasing incidence continued till 1985 and was due to insufficient detection of cases and reduction of DDT coverage. The situation had been further aggravated by the settlement of 30,000 Afghan refugees from malaria endemic areas of the country. This trend was reversed in the subsequent years [6].

### Malaria resurgence (1991 onwards)

In 1991–1992, as a result of armed conflict and social unrest in the newly-independent Tajikistan, the functioning of the health services including anti-malarial activities had reduced almost to a standstill. Marked changes in agricultural practices, particularly the increase in the cultivation of rice and acute shortage of agricultural pesticides for cotton production, had led to an increase in vector breeding grounds and vector densities. This was further exacerbated by a massive (estimated 600,000 persons) return of Tajik refugees in 1993 from malaria endemic areas of Pakistan and Afghanistan. As a result, large-scale malaria epidemics occurred during 1993–1997. Malaria cases were registered on the territory of 62 of the total 64 districts in the country [7. Indigenous cases of *P. falciparum* occurred for the first time since the 1940s.

Another peculiarity was a remarkable change in the ratio of *P. vivax* with long incubation to *P. vivax* with short incubation. Unlike in the pre-elimination era, when it was estimated to represent 10% of cases, malaria with long incubation constituted about 40% of total *P. vivax* cases [8]. The situation was similar to that in Azerbaijan and Northern Afghanistan during the 1970s [9, 10]. Recent findings in India indicate that up to 20% of total *P. vivax* cases in Eastern and Central India is due to parasites with long latency [11–13]. The changing ratio suggests the possibility that *P. vivax* strains with a long incubation, such as a long period of latency (e.g. 16–18 months) may delay the completion of malaria elimination beyond the expected target date [10].

Although official statistics indicated total of 29,794 malaria cases occurred in 1997, this was only a small minority of the total and an expert’ assessment was that there was at least total of 400,000 cases in the country, considerably in excess of malaria incidence during pre-elimination years. *Plasmodium falciparum* malaria constituted up to 5% of total malaria cases, the major foci were confined to central and southern districts.

### Organization of the response

In response to the malaria emergency, the World Health Organization (WHO), both at the Headquarters in Geneva and in the WHO European Region, assisted the Government of the Republic in 1997 in the development of the malaria emergency plan of action for the containment of large-scale malaria epidemics. The WHO also provided the country with emergency stocks of anti-malarial drugs and insecticides. The plan of action document constituted the basis of the WHO appeal to various governmental, non-governmental and to the international organizations to assist the Government of Tajikistan in the fight against malaria.

The response to the appeal was very successful. By 1998, considerable financial, scientific and in-kind support was provided by the WHO, UNICEF, European Commission Humanitarian Aid Office, FAO, USAID, Merlin, and the Agency for Technical Cooperation and Development (ACTED), as well as financial aid from the Governments of Japan, Italy, Norway, and this played a crucial role in malaria epidemic control.

This assistance was instrumental in the re-establishment of a specialized malaria control programme in Tajikistan, including the central, 4 regional (*oblast*) and 15 district malaria control centers the staff of which was responsible for planning, implementation and assessment of the efficacy of anti-malaria measures.

In 1997, the number of reported malaria cases reached its peak, when nearly 30,000 cases were registered. In the following years, the total number of cases dramatically fell due to implemented anti-malarial interventions, but the number of confirmed *P. falciparum* cases increased, showing 831 cases in 2000, as against 183 cases in 1997. Thus, the main concern was to prevent expansion of *P. falciparum,* both in terms of territory and incidence.

### Establishment of a *Plasmodium falciparum* elimination programme

Following the recommendations of the WHO [14] in 2006, the Government decided to embark upon interruption of *P. falciparum* malaria local transmission on a priority basis. The programme of *P. falciparum* elimination was financially supported by the Global Fund for control of AIDS, Tuberculosis and Malaria. The target date was selected to be 2010. The elimination strategy was based on a thorough knowledge of peculiarities of epidemiology of *P. falciparum* malaria in Tajikistan.

### Epidemiology of *P. falciparum* malaria in Tajikistan

**Fig. 2:**
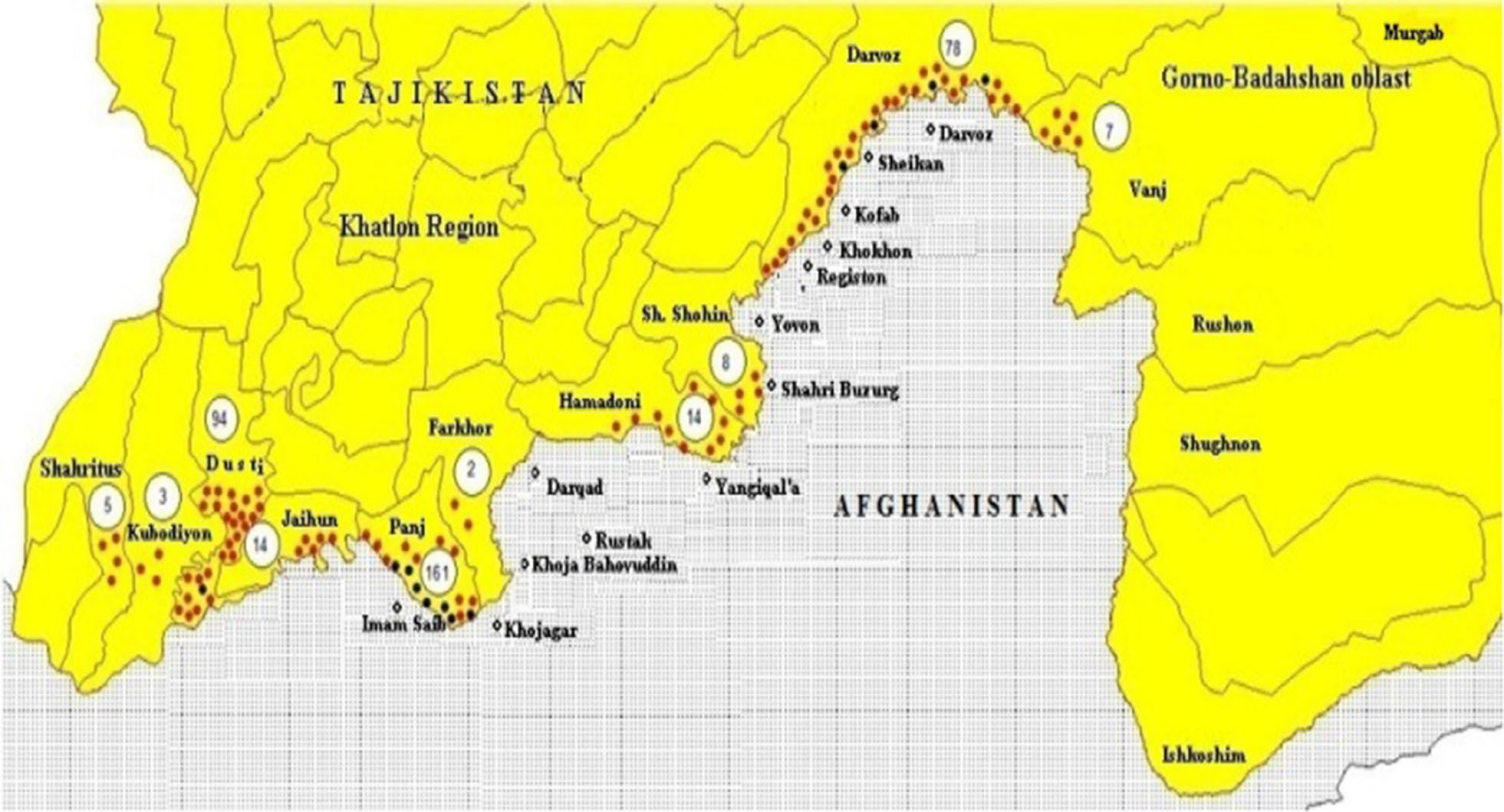
*Plasmodium falciparum* cases and foci in border areas of Tajikistan and Afghanistan, 1994–2008. *Black dots* districts with outbreaks; *red dots* districts with sporadic cases; *figures in circles* total number of cases [8]

From 1994 to 2008, a total of 3585 indigenous cases of *P. falciparum* were registered in 28 administrative districts and in 3 cities (Dushanbe, Kulob and Kurgan-Tyube). Thus, a total of 1.9 million people had been at risk of *P. falciparum* malaria (about 26% of the total population of the Republic) [15]. The 13 districts of Khatlon region situated in a close proximity to the territory of Afghanistan, constituted the first group with the highest *P. falciparum* risk (76% of the total cases) (Fig. 2).

The malaria risk depended upon several epidemiological factors. First, there is a movement of population from Tajikistan to border areas of Afghanistan, which is facilitated by the recent construction of five bridges over the river Punj. The river is not sufficiently broad (between 200 and 900 m wide) to prevent infected Anopheles mosquitoes flying over it. Second, the riverine districts are suitable for agriculture. The main crop is rice, resulting in favourable conditions for breeding of the two principal malaria vectors in Tajikistan—*Anopheles pulcherrimus* and *Anopheles superpictus*—and a secondary malaria vector, *Anopheles hyrcanus*. Third, the season of malaria transmission was about 6 months allowing local transmission of both *P. vivax* and *P. falciparum*. Simultaneous local transmission of both malaria species was observed in active malaria foci.

Several districts of central Tajikistan constituted the second group with about 18% of total *P. falciparum* cases. The season of transmission is shorter than 6 months and less conducive for local transmission for *P. falciparum*. Transmission is due to *An. superpictus,* with some role played by *Anopheles claviger*.

The third group consisted of a few districts in the northern Tajikistan reporting only sporadic cases of indigenous *P. falciparum* malaria, but potentially posing a serious problem in case of introduction of imported cases either from inside of the country or from abroad. The natural conditions for re-establishment of malaria transmission are rather favorable due to the presence of several efficient vectors including *An. superpictus, Anopheles martinius, Anopheles artemievi*.

### Seasonal pattern of *P. falciparum* transmission in Tajikistan

The longest season of malaria transmission is in the plains (normally about 6 months), during April till September–October, followed by hills—about 5 months, and shortest is in the mountains—3 to 4 months. The principal vector in the plains of the southern Tajikistan is *An. pulcherrimus*, followed by *An. hyrcanus* and to lesser extent by *An. superpictus*. The latter is a principal malaria vector in the hills and mountains although it can breed in the plains as well. The main breeding sites for *An. pulcherrimus* and *An. hyrcanus* are rice fields, while *An. superpictus* breeds in small collections of water along the river beds [4].

**Fig. 3:**
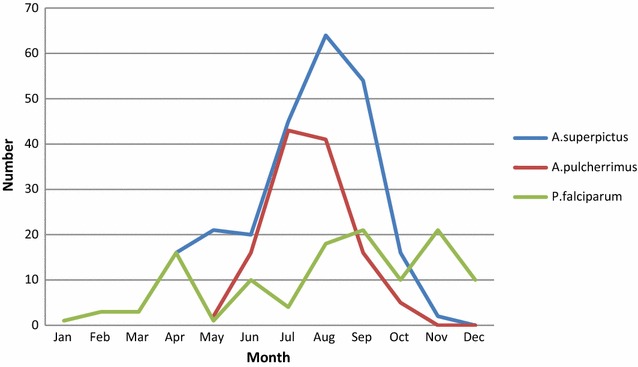
Seasonal pattern of *Anopheles pulcherrimus*, *Anopheles superpictus* and number of *Plasmodium falciparum* cases, Tajikistan, 2005–2008

Seasonal pattern of density of principal vectors in the southern and central Tajikistan is different. The peak of *An. pulcherrimus* is in July, while that of *An. superpictus* is in September [4] (Fig. 3).

In the northern Tajikistan, the principal vector was *An. superpictus*, followed by *An. artemievi*. The latter is a sibling species of *An. martinius* from the *Anopheles maculipennis* group. The ecology of the principal vectors is also different. While *An. pulcherrimus* is a semi-exophilic species, *An. superpictus* is endophilic and endophagic, preferring to feed on humans than animals. It can also infect during the diapause (the period of reproductive inactivity). The cases of *P. falciparum* during the late autumn (as shown in Fig. 3), might result from infection by diapausing mosquitoes.

*Anopheles superpictus* is a very good malaria vector, as illustrated by the results of studies on sporozoite index; 0.28% in mosquitoes caught in the wild (1/360) and 2.3% (3/128) inside dwellings [4]. The presence of both principal vectors might facilitate a “relay” transmission of infection and may have resulted in the establishment of malaria foci with both malaria species—*P. vivax* and *P. falciparum* delaying the arrest of local malaria transmission. It was found that *P. falciparum* foci could be eliminated within 1–2 years, while 3–4 years was necessary to achieve the same goal in case of “mixed” foci [16]. In southern Tajikistan, almost 80% of all malaria foci were mixed [6]. Differences in the phenology and behaviour of principal vectors had determined the choice of different approaches to vector control interventions.

### Eco-epidemiological types of *P. falciparum* malaria

There are two eco-epidemiological types of *P. falciparum* in Tajikistan. The urban eco-type constituted 19% of the total malaria cases in the Republic, the rest being rural. *P. falciparum* malaria occurred in three cities, including the capital of the country—Dushanbe, where it was associated with the secondary malaria vector—*An. claviger* whereas in the other two cities—Kulyab and Kurgan-Tyube it was due to *An. pulcherrimus* and *An. superpictus,* respectively [16].

### Occupation and age related to *P. falciparum* malaria

*Plasmodium falciparum* malaria affected different age groups almost equally—pattern typical for epidemic malaria. There was not particular preponderance for any occupational category.

### Susceptibility of *P. falciparum* to anti-malarial drugs

Studies undertaken in the Republic at the beginning of the 2000s revealed the presence of *P. falciparum* resistance at the RI–RII levels to chloroquine [7]. Artesunate plus sulfadoxine and pyrimethamine replaced chloroquine and demonstrated efficacy of almost 100% [8].

It appeared that introduction of the ACT had played an important role in the reduction of malaria incidence (Additional file 1).

### Susceptibility status of Anopheles mosquitoes to various insecticides

The principal malaria vectors in Tajikistan are resistant to DDT, but fully susceptible to malathion and pyrethroids. These have being in use since 1998 [7].

### Organization of the *P. falciparum* malaria elimination programme in Tajikistan

#### Malaria surveillance system

During the years of malaria elimination (1950–1960) in Tajikistan, there was a network of “malaria stations”, the personnel of which was responsible for planning, organization/implementation and evaluation the efficacy of anti-malaria interventions. The latter were carried out jointly with the basic health services and with participation of municipalities and community members. The scope of malaria surveillance system during that period consisted of capacity building, large-scale use of the IRS, supported by anti-larval measures, selective use of MDA, case detection and treatment activities jointly with the staff of health treatment facilities. A network of parasitological laboratories with reference laboratories at the central and regional levels ensured timely detection and treatment of cases.

At present, an overall responsibility for the organization, implementation and evaluation of anti-malarial activities is with the State Sanitary Epidemiological Board of the Ministry of Health and Social Welfare at the central, provincial and district levels. Responsibility for malaria surveillance was delegated to the Republican Centre for the control of tropical diseases at the central and the peripheral levels. The scope of malaria surveillance system is presented in an organigram (Additional file 2).

Medical and paramedical staff in the governmental sectors-engaged in the curative activities at various levels are responsible for detection and treatment of malaria cases in accordance with the recommendations of the national protocol of anti-malarial treatment.

Malaria is a notifiable disease in the Republic. Each detected case of malaria must be immediately reported to territorial centre of control of tropical diseases or to the territorial office of the State Sanitary Epidemiological Board. In addition, details of the case, including diagnosis and the patient’s address are sent to the Central District Hospital.

Following the receipt of a report of malaria case, a group of specialists (epidemiologist, entomologist, and laboratory technician) at district level is despatched to the malaria focus to carry out an epidemiological investigation and classification of case and focus. In case of malaria outbreak, a special mobile team, consisting of epidemiologist, entomologist and laboratory specialist at the central and regional levels is sent for investigation and organization of anti-malarial interventions.

Selected indicators employed by malaria surveillance had been thoroughly monitored and evaluated. Among epidemiological indicators following were selected—ABER, morbidity, SPR, SFR. Entomological indicators included seasonal density of primary and secondary vectors, densities of larva, and larva density dynamics in treated breeding sites. Operational indicators included total coverage by the ITNs, percentage (%) of its use last night, the IRS targeted population against actual protected population, time lag between detection of case and laboratory diagnosis, percentage of treated cases within 24 h following laboratory confirmed diagnosis.

Each and every *P. falciparum* malaria focus was epidemiologically investigated and classified (new active, residual active, residual non-active, potential and cleared) and included into the national register of malaria foci of the country. For laboratory confirmed *P. falciparum* malaria cases age, sex, and occupation were documented and each was epidemiologically classified as indigenous, imported, introduced or recrudescence.

The GIS and GPS techniques were used for malariological stratification of the territory of Tajikistan. It was based on of the type of landscape, climate characteristics and occupational activities of the local population. Principal and secondary malaria vectors had been identified previously, their habitats, their seasonal epidemiology and their insecticide resistance status characterized. Breeding sites for malaria vectors had been identified, mapped, measured and included into the national roster of malaria vector breeding sites. Creation of the national roster facilitated planning process for anti-larval measures and procurement purposes. Seasonal patterns of the adult and larval densities of the Anopheles mosquitoes were monitored regularly in the sentinel sites. The beginning and the end of the malaria transmission season was determined during 2004–2007 in different strata through the use of the Moshkowski method [7].

Detection of malaria cases was carried out by the staff of the basic health services. Both active (ACD) and passive case detection (PCD) mechanisms were deployed. The frequency of the ACD (house to house visits) during the malaria transmission season was 7 days. Epidemiological investigations and classification of malaria cases and malaria foci were carried out by the staff of specially formed mobile teams. The scope of the team included also the organization and implementation of special surveys both during and outside malaria transmission season, as well as reactive case detection (RCD).

The principal method of detection of malaria parasites was microscopy of collected blood slides using Giemsa staining of thin and thick smears. In accordance with the standard WHO recommendations [14], several surveys were undertaken to evaluate the efficacy and safety of implemented anti-malarial interventions.

To ensure the safety of a single dose (30 mg) of primaquine as gametocytocidal drug in the treatment of a *P. falciparum* case, a special survey was conducted among the local population in malaria foci. The dye reduction method was used for qualitative screening for G6PD deficiency in population in malaria foci. The average prevalence was found to be rather low (2.1% with variations from 0.8 to 4%) which ensured safety of primaquine use [17].

Excellent efficacy of ACT (using artesunate and SP) for the treatment of *P. falciparum* was demonstrated in the treatment of 2686 cases [18].

Studies on the longevity of the residual activity of various pyrethroids (Icon, Zolfac, Tryton, alfacypermethrin) had been carried out in two districts of Hatlon region, using standard techniques recommended by the WHO [14]. Epidemiological impact of the use of pyrethroids had been evaluated in total of 28 districts of the Republic, before and after spraying operations.

Efficacy of the use of the larvivorous fish *Gambusia affinis* in conjunction with other anti-malarial measures was carried out in 6 districts of Tajikistan. Epidemiological efficacy of the LLIN was carried out in 3 districts of Tajikistan. Malaria KAP studies were carried out in 6 districts of the country among 1009 persons, representing 5% of the targeted population.

### Implementation of the activities of *P. falciparum* programme

Epidemiological classifications of *P. falciparum* malaria foci served as a basis for the selection of various interventions. Four epidemiological types of *P. falciparum* malaria focus were adopted in Tajikistan: (a) potential, (b) new active, (c) residual active, (d) cleared up [14]. The repertoire of selected anti-malarial measures is presented in the Tables 1 and 2.

**Table 1:** Case detection and treatment in different epidemiological types of *Plasmodium falciparum* foci, Tajikistan, 1994–2009

Epidemiological type of *P. f* focus	Case detection and treatment					
	Active case detection*, including re-active case detection	Passive case detection	Presumptive treatment**	Mass seasonal prophylaxis***	Radical treatment	Mass blood survey****
Potential	+	+	+	−	+	−
New active	+	+	+	+	+	+
Residual active	+	+	+	+	+	+
Cleared up	−	+	−	−	−	+

* ACD at 7 day frequency; ** till 2002 only; *** selectively only; **** selectively only

**Table 2:** Vector control activities in different epidemiological types of *Plasmodium falciparum*, Tajikistan, 1994–2009

Epidemiological type of *P. f* focus	Entomological observations	Indoor residual insecticide spraying	Insecticide impregnated mosquito nets	Biological larviciding (Gambusia fish)	Environmental management	Insecticide dispensers
Potential	+	−	−	+	+	+
New active	+	+	+	+	+	+
Residual active	+	+	+	+	+	+
Cleared	+	−	−	+	+	+

Active case detection (ACD) was the principal mechanism of detection of malaria cases, supported by reactive case detection (RCD) and passive case detection (PCD). Adopted frequency of ACD at 7 day interval had resulted in early detection of cases (Additional file 3).

Annual blood examination rate (ABER) was very close to stipulated 10% ensuring detection of cases in time and space (Table 3).

**Table 3:** ABER, Tajikistan, 2006–2010

	2006	2007	2008	2009	2010
Total slides collected	175,894	159,232	159,068	165,266	173,523
ABER %	8.99	8.14	8.13	8.46	8.88

All treated malaria cases were laboratory confirmed usually by microscopy within 24 h. Time-lag between detection of case and the beginning of treatment did not exceed 3 days in 90% of cases. This was facilitated by well-organized malaria laboratory services of the Republic. The network of laboratory service comprised of 4 regional and 244 peripheral malaria laboratories. It consisted of one Central Reference Laboratory, responsible for training and refresher training of the malaria laboratories staff in the country, as well as for re-checking all positive and 10% of all negative slides. All laboratories were provided with workable microscopes, reagents and alike. All sanctioned posts of laboratory technicians were filled by the technicians well trained in malaria diagnosis.

All patients were treated at the health treatment facilities as inpatients. Most treated cases (84%) were uncomplicated *P. falciparum* malaria [18]. Only 2 fatal cases of *P. falciparum* malaria were registered in two young children due to a very late admission to health treatment facility [18].

The choice of vector control interventions and their timing relied upon the results of entomological observations carried out regularly by the network of 25 regional and district tropical diseases centers in Tajikistan, each with one entomology section. The latter is staffed with 1–2 assistants of the entomologist, responsible for conducting entomological observations in sentinel sites. The scope of entomological surveillance included monthly collection of data on: (i) Anopheles species composition; (ii) dynamics of adult mosquito populations (both endophilic and exophilic)—once in 10 days; (iii) man-biting densities (human landing catch technique); (iv) dynamics of larva populations in breeding sites.

In addition, the following were documented: (i) external air temperature; (ii) inventory of breeding sites; (iii) tests on susceptibility of Anopheles mosquito to various insecticides; (iv) efficacy of the use of larvivorous fish, LLIN and IRS; (v) residual activity of insecticides on sprayed surfaces; (vi) establishment of a sporozoite index.

Indoor residual insecticide spraying (IRS) was one of the main interventions in all malaria foci. Until 2001, the insecticide in use was Malathion. Unfortunately, the acceptance by the population was rather low mainly due to its odour and need for repeat applications during the season of transmission.

Since 2001, various pyrethroids were used. Before implementation on a large scale, studies on longevity of their efficacy under local climatic conditions were undertaken [8]. It was found that the residual activity of alfacypermethrin (5% sp) was 82% after 2.5–3.0 months following spraying [19]. Before the *P. falciparum* elimination programme was launched in 2006, only one cycle of the IRS with pyrethroids was possible due to shortage of funds and it was directed towards priority reduction of *P. vivax* cases, which constituted about 95% of total cases.

**Fig. 4:**
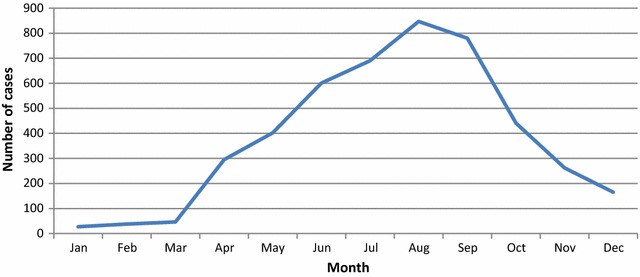
Seasonal pattern of *Plasmodium vivax* cases, Tajikistan, 2005

*Plasmodium vivax* malaria cases begin to present in April (Fig. 4). These represent either the primary illness of a long incubation or a relapse of short incubation period *P. vivax*. Beginning of June marks the appearance of fresh *P. vivax* cases with short incubation period, with the peak in July–August. One cannot exclude, however, that these are still cases of long latency *P. vivax* with an early primary illness (rather like the Madagascar or St Elizabeth strains) (White, pers. comm., 2016). Therefore, to prevent/reduce the local transmission of malaria, the application of the IRS would have to be carried out not later than end of April-beginning May. Thus, the maximal efficacy of the first cycle would last till the end of July-beginning of August, abruptly diminishing thereafter. The peak of *P. falciparum* transmission is during the months of August–September, a period where residual activity of the insecticide is minimal. This explained the reason of continuous transmission of *P. falciparum*.

The situation completely changed with implementation of the *P. falciparum* elimination programme. Two rounds of the IRS in all foci in 2007–2008 (Additional file 4) changed the epidemiological scenario dramatically, contributing to interruption of *P. falciparum* malaria transmission. More than 1.5 million people were protected by the IRS during 2006–2008. The protective coverage of the targeted population varied between 90 and 95% [8]. Households were considered fully protected only if 100% of living rooms had been sprayed.

All WHO recommendations with regard to safety measures had been followed strictly. Different kind of pyrethroids had been used—Triton in 2006, alphacypermethrine in 2007–2008. Use of the ITNs was considered as complementary measure. During the period 2000–2008, total of 137,000 insecticide impregnated nets were distributed (Additional file 5). Until 2006, insecticide-treated nets (ITNs) were distributed to families having at least one child less than 5 years of age. Primigravidae women were also included into priority list. Since 2006, with the arrival in the country of the LLINs, additional priorities for distribution were as follows: (a) populations living in areas with the highest levels of *P. falciparum* transmission, (b) populations living in *P. falciparum* malaria foci close to the border with Afghanistan.

Overall, 98.1% of targeted households had one mosquito net. Studies on the actual use of the mosquito nets had revealed that 71.1% of the owners used them every night [7, 8]. Washing the LLIN once a year revealed 100% efficacy during subsequent 3-year period [20].

Another complementary intervention was the use of the larvivorous fish, *Gambusia affinis*. It started in 1999 with the support of the ACTED till 2006. Annually, fishes were placed into breeding sites of Anopheles mosquitoes with the total surfaces of 37 ha. With the commencements of *P. falciparum* malaria elimination activities, targeted surfaces were expanded up to 795 ha annually. Efficacy of the use of larvivorous fish was evaluated in terms of absence of pupa and larva of 3–4 instars [3, 21] (Additional file 6).

In 2000–2008, more than one million of people, including 150,000 school children, who were residents of malaria foci were subjected to health education activities. The latter included the distribution of booklets, pamphlets, leaflets and alike on prevention of malaria. Mass media (newspapers, radio, television etc.) played a very important role in creation of malaria awareness among the population and establishment of an atmosphere of cooperation with the health services.

On the whole, it could be safely said that the population under malaria risk in the Republic demonstrated a positive attitude towards implemented anti-malarial activities and on many occasions rendered cooperation to the health services staff. A number of courses and seminars had been organized for health personnel of different categories both in the governmental and private sectors on malaria diagnosis, treatment and epidemiology. In total 305 laboratory technicians were trained/refresher trained on malaria diagnosis. 1549 physicians, epidemiologists, parasitologists and entomologists benefitted from malaria orientation training.

17 specialists from public health side participated in the international training courses on malaria, organized by the WHO.

Border malaria related activities are a very important component of the malaria elimination programme of Tajikistan. The Republic of Tajikistan has a direct agreement with Afghanistan to exchange information on various aspects of malaria prevention and control in border areas between the two countries. This has been done through the WHO Eastern Mediterranean Regional Office and the WHO European Regional Office. The two countries had also agreed to carry out the IRS simultaneously with the use the same insecticides on both sides of the border. A number of Afghani health workers were trained in Tajikistan in malaria-related activities. This cooperation proved to be very fruitful for both sides—considerable reduction of malaria problem in border areas of Afghanistan and interruption of local transmission of *P. falciparum* in Tajikistan.

Financial support for the *P. falciparum* elimination programme (2006–2010) came from two important sources—the Government of Tajikistan and the Global Fund for control of AIDS, tuberculosis and malaria. Although the Governmental contribution was less than that by the Global Fund, it demonstrated an increasing trend every year (Additional file 7).

### Achievement of the elimination of *P. falciparum* in Tajikistan

**Fig. 5:**
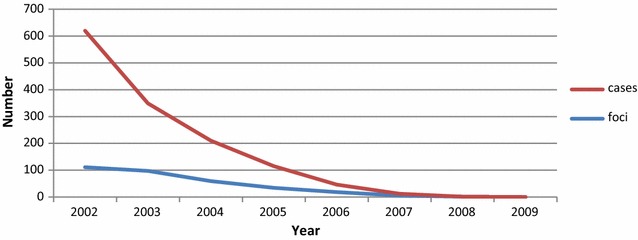
Achievement of interruption of local *Plasmodium falciparum* malaria transmission in Tajikistan

The goal of interruption of local *P. falciparum* malaria transmission was achieved in 2009 one year earlier than it was originally envisaged (Fig. 5). Since 2009, only a few imported cases of *P. falciparum* malaria were reported in the Republic. Continued routine activities of the malaria surveillance system did not discover indigenous cases of *P. falciparum* in the following years since interruption of transmission. In 2012, selective mass fever surveys among the population had been carried out in the erstwhile active *P. falciparum* foci in 4 districts of the southern Tajikistan. These were negative.

### Role of *P. falciparum* elimination programme in reduction of *P. vivax* problem

Evidence exists that the achievements of the programme facilitated the reduction of *P. vivax* malaria incidence in Tajikistan. There are several factors which positively contributed to the process. Introduction of the IRS second cycle prevented the contraction of *P. vivax* during the whole period of malaria transmission. Adoption of the 7-day frequency of house-to-house visits resulted in an early detection, diagnosis and prompt treatment of cases. Vector control complimentary measures, particularly the use of larvivorous fishes considerably reduced the population of semi-exophilic *An. pulcherrimus* mosquitoes—one of the principal vectors of *P. vivax* in Tajikistan.

As a result, the last indigenous case of *P. vivax* was reported in Tajikistan in 2013 (Additional file 8). At present, the National Malaria Elimination Programme of Tajikistan is in the process of preparation for the WHO Certification of malaria elimination in the Republic.

### Prevention of malaria re-introduction into malaria free territories of Tajikistan

Following elimination of *P. falciparum* malaria, the national malaria programme developed a map of the risk of malaria re-introduction in Tajikistan based on the assessment of receptivity and vulnerability [3, 22]. This map may serve as a basis for implementation of malaria preventive activities (Additional file 9).

### Factors contributing towards elimination of *P. falciparum* malaria in Tajikistan

The reasons for such a success were as follows.

#### Political commitment

The goal of *P. falciparum* malaria elimination was considered as an important factor contributing towards the national plan of socio-economic development by preventing incidence and mortality from the disease. This goal was clearly reflected in the Governmental decree No. 502 of 30.12.2005 “Programme of Control of Tropical Diseases (malaria) in the Republic of Tajikistan, 2006–2010”. The establishment of the National Committee at the Central Government level for coordination control measures against AIDS, tuberculosis and malaria played an important role. It was headed by the representative of the Government of Tajikistan with members being from various ministries, the NGOs, private sector, and religious leaders.

#### Partnership

The National Malaria Programme had enjoyed albeit limited, but continuous support right from its inception in 1993–1994 being it of international or national source. Several governmental, non-governmental and international organizations assisted the programme in the development and implementation of the activities.

#### Sound strategy

The strategy developed was based on a good knowledge of the peculiarities of epidemiology of malaria in various parts of the country facilitated by the national and international institutions. This knowledge allowed the programme to select the anti-malarial interventions, which were most appropriate under local conditions.

#### Holistic approach

Anti-malaria interventions were carried in combination. Most crucial interventions which had been instrumental in the arrest of local *P. falciparum* transmission was use of pyrethroids for IRS, adjusted frequency of the IRS, long-lasting insecticide-impregnated mosquito nets, deployment of ACT in combination with a single dose of primaquine, use of biological larviciding.

#### Community participation

Although community members had not been directly involved in the implementation of various anti-malarial activities, their role was important in the acceptance of all carried out interventions by the programme and adherence to all its recommendations in respect of personal protection measures. This attitude was reflected in high coverage by the IRS, ACT and ITNs.

#### Border malaria

Reduction of local malaria transmission in border areas in Afghani sites had contributed to decrease of malaria on Tajikistan site as well. Imported cases from the Northern Afghanistan had been nearly all *P. vivax* cases.

## Conclusions

Elimination of *P. falciparum* in Tajikistan did not eliminate the threat of its importation from the countries with continuous transmission of malaria, particularly from the countries of South and South-East Asia in general and from Afghanistan in particular. Therefore, there is a need to continue to carry out anti-malaria preventive measures in the Republic, particularly in border areas within the framework of the agreement on cooperation between two countries.

To meet the challenge of the prevention of re-establishment of local transmission of *P. falciparum* malaria, the National Malaria Programme of Tajikistan has created a reserve stock of anti-malarial drugs, insecticides and ITNs at the central and district levels. Deployment of biological larviciding (*Gambusia affinis*) continues. Capacity building activities continues on the same scale including health education activities among population residing in areas with high and moderate malaria potential.

## Additional files

**Additional file 1.** Impact of the use of ACT on *P.falciparum* incidence, Tajikistan, 2004–2009.

**Additional file 2.** Malaria surveillance system, Tajikistan.

**Additional file 3.** Detection of *P.falciparum *cases by ACD, Tajikistan, 2004–2007.

**Additional file 4.** Use of the IRS in foci (people protected), Tajikistan, 2006–2008.

**Additional file 5.** Distribution of the ITNs/LLIN, Tajikistan, 2006–2010.

**Additional file 6.** Impact of *Gambusia affinis* on density of *Anopheles* larva, Tajikistan, 2007.

**Additional file 7.** Financial support of the *P.falciparum* elimination programme, Tajikistan, 2006–2010 (US$).

**Additional file 8.** Dynamics of *P.vivax* and* P.falciparum* cases, Tajikistan, 1997–2012.

**Additional file 9.** Potential risk of re-introduction of malaria in Tajikistan.

## Abbreviations

ABER: annual blood examination rate
ACD: active case detection
ACT: artemisinin-based combination therapy
ACTED: Agency for Technical Cooperation and Development
AS/SP: artesunate plus sulfadoxine-pyrimethamine
CQ: chloroquine
DDT: dichloro-diphenyl-trichloroetane
FAO: World Food Organization
GIS: geographic information systems
GPS: global position systems
ITN: insecticide-treated net
IRS: indoor residual house spraying
LLIN: long-lasting insecticide-treated net
PCD: passive case detection
PCR: polymerase chain reaction
RCD: reactive case detection
RDT: rapid diagnostic test
SFR: slide *P. falciparum* positive case
SPR: slide positive rate
SP: sulfadoxine/pyrimethamine
UNICEF: United Nations’ Children Fund
USAID: United States Agency for International Development
USSR: Union of Soviet Socialist Republics
WHO: World Health Organization
