# Supplementary material for: Elimination of Plasmodium falciparum malaria in Tajikistan
Source: Malar J. 2017 May 30;16:226. doi: 10.1186/s12936-017-1861-5 (PMC5450305; doi:10.1186/s12936-017-1861-5)
Supplement: Supplementary file 1 — Additional file 1. Impact of the use of ACT on P.falciparum incidence, Tajikistan, 2004–2009. [file 12936_2017_1861_MOESM1_ESM.docx]

**Impact of the use of ACT on P.falciparum incidence, Tajikistan, 2004-2009**
