# Supplementary material for: Elimination of Plasmodium falciparum malaria in Tajikistan
Source: Malar J. 2017 May 30;16:226. doi: 10.1186/s12936-017-1861-5 (PMC5450305; doi:10.1186/s12936-017-1861-5)
Supplement: Supplementary file 3 — Additional file 3. Detection of P.falciparum cases by ACD, Tajikistan, 2004–2007. [file 12936_2017_1861_MOESM3_ESM.docx]

**Detection of *P.falciparum* cases by ACD , Tajikistan, 2004-2007**

|  | 2004 | 2005 | 2006 | 2007 |
| --- | --- | --- | --- | --- |
| Total *P f* cases | 152 | 81 | 28 | 7 |
| Detected by ACD | 105 | 53 | 19 | 7 |
| ACD %% | 69.1 | 65.4 | 67.8 | 100.0 |
