# Supplementary material for: Elimination of Plasmodium falciparum malaria in Tajikistan
Source: Malar J. 2017 May 30;16:226. doi: 10.1186/s12936-017-1861-5 (PMC5450305; doi:10.1186/s12936-017-1861-5)
Supplement: Supplementary file 4 — Additional file 4. Use of the IRS in foci (people protected), Tajikistan, 2006–2008. [file 12936_2017_1861_MOESM4_ESM.docx]

**Use of the IRS in foci (people protected), Tajikistan, 2006-2008**
