# Supplementary material for: Elimination of Plasmodium falciparum malaria in Tajikistan
Source: Malar J. 2017 May 30;16:226. doi: 10.1186/s12936-017-1861-5 (PMC5450305; doi:10.1186/s12936-017-1861-5)
Supplement: Supplementary file 5 — Additional file 5. Distribution of the ITNs/LLIN, Tajikistan, 2006–2010. [file 12936_2017_1861_MOESM5_ESM.docx]

**Distribution of the ITNs/ LLIN, Tajikistan, 2006-2010**
