# Supplementary material for: Elimination of Plasmodium falciparum malaria in Tajikistan
Source: Malar J. 2017 May 30;16:226. doi: 10.1186/s12936-017-1861-5 (PMC5450305; doi:10.1186/s12936-017-1861-5)
Supplement: Supplementary file 6 — Additional file 6. Impact of Gambusia affinis on density of Anopheles larva, Tajikistan, 2007. [file 12936_2017_1861_MOESM6_ESM.docx]

**Impact of *Gambusia affinis* on density of Anopheles larva, Tajikistan, 2007.**
