# Supplementary material for: Elimination of Plasmodium falciparum malaria in Tajikistan
Source: Malar J. 2017 May 30;16:226. doi: 10.1186/s12936-017-1861-5 (PMC5450305; doi:10.1186/s12936-017-1861-5)
Supplement: Supplementary file 7 — Additional file 7. Financial support of the P.falciparum elimination programme, Tajikistan, 2006–2010 (US$). [file 12936_2017_1861_MOESM7_ESM.docx]

**Financial support of the P.falciparum elimination programme, Tajikistan, 2006-2010 (US$)**
