# Supplementary material for: Elimination of Plasmodium falciparum malaria in Tajikistan
Source: Malar J. 2017 May 30;16:226. doi: 10.1186/s12936-017-1861-5 (PMC5450305; doi:10.1186/s12936-017-1861-5)
Supplement: Supplementary file 8 — Additional file 8. Dynamics of P.vivax and P.falciparum cases, Tajikistan, 1997–2012. [file 12936_2017_1861_MOESM8_ESM.docx]

**Dynamics of *P.vivax* and *P.falciparum cases*, Tajikistan, 1997-2012**
