# Supplementary figures and images for: Elimination of Plasmodium falciparum malaria in Tajikistan
Source: Malar J. 2017 May 30;16:226. doi: 10.1186/s12936-017-1861-5 (PMC5450305; doi:10.1186/s12936-017-1861-5)

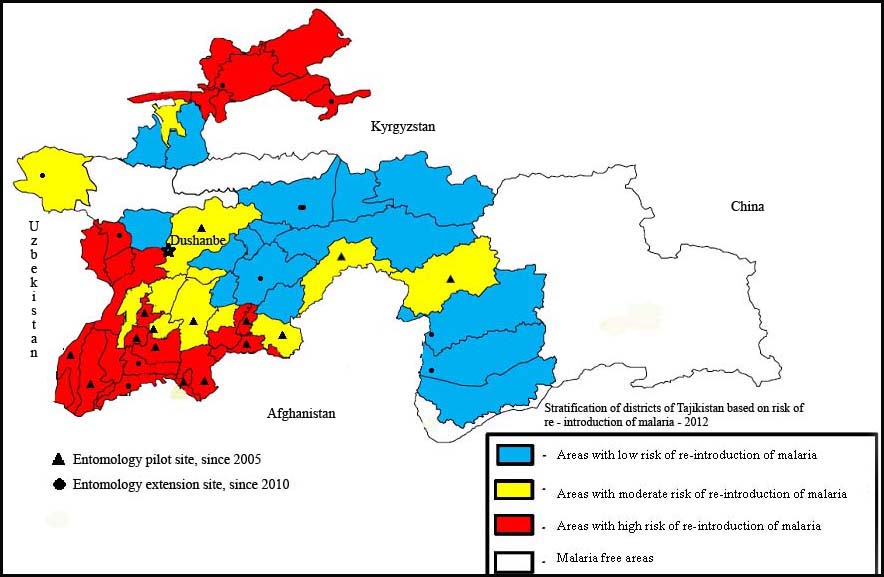


**Potential risk of re-introduction of malaria in Tadjikistan.**

Supplement: Supplementary file 9 — Additional file 9. Potential risk of re-introduction of malaria in Tajikistan. [file 12936_2017_1861_MOESM9_ESM.docx]
