# Supplementary material for: Elimination of Plasmodium falciparum malaria in Tajikistan
Source: Malar J. 2017 May 30;16:226. doi: 10.1186/s12936-017-1861-5 (PMC5450305; doi:10.1186/s12936-017-1861-5)
Supplement: Supplementary file 9 [file 12936_2017_1861_MOESM2_ESM.docx]

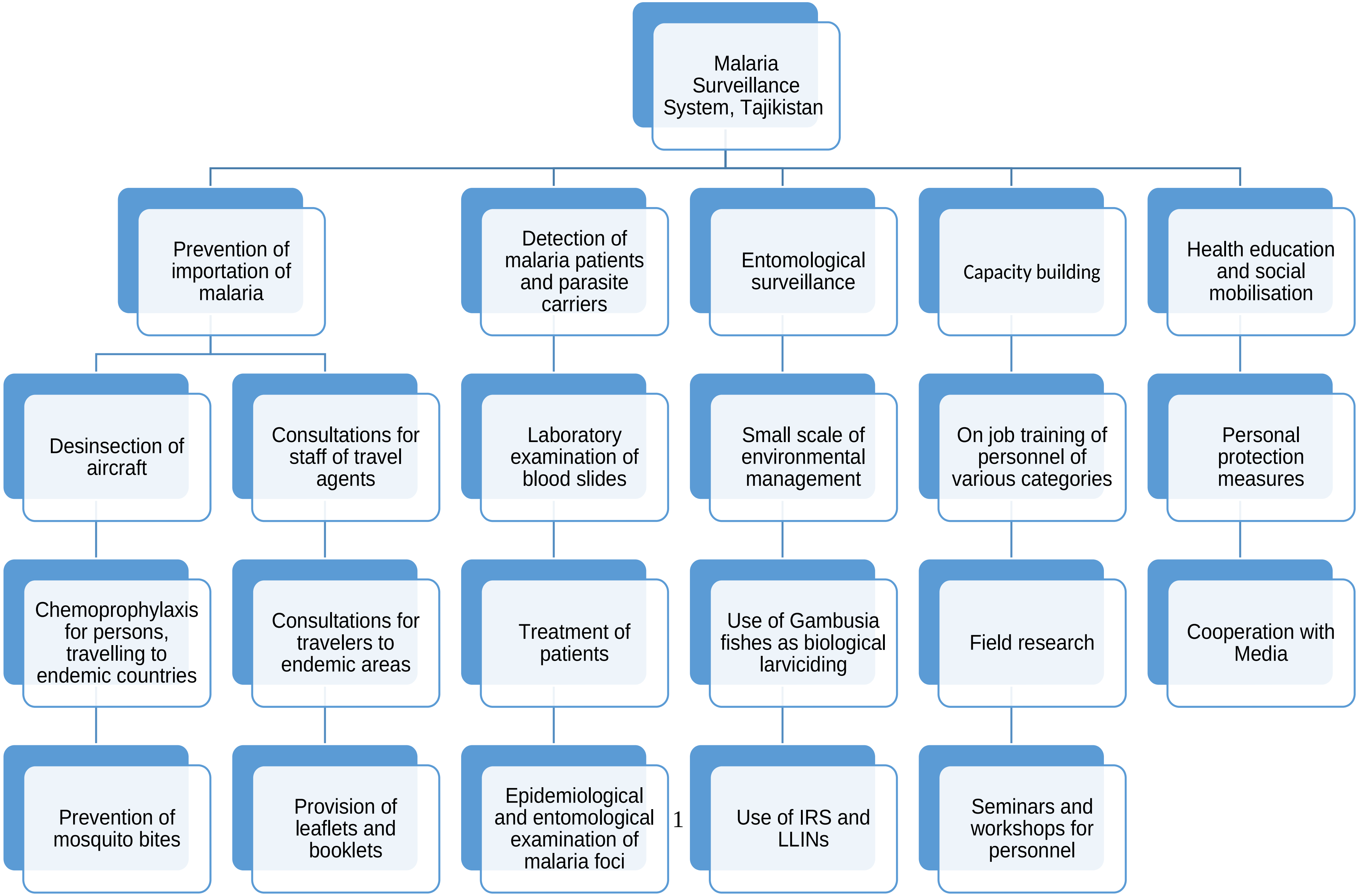
Malaria Surveillance System, Tajikistan
Prevention of importation of malaria
Detection of malaria patients and parasite carriers
Entomological surveillance
Capacity building
Health education and social mobilisation
Desinsection of aircraft
Consultations for staff of travel agents
Laboratory examination of blood slides
Small scale of environmental management
On job training of personnel of various categories
Personal protection measures
Chemoprophylaxis for persons, travelling to endemic countries
Consultations for travelers to endemic areas
Treatment of patients
Use of Gambusia fishes as biological larviciding
Field research
Cooperation with Media
Prevention of mosquito bites
Provision of leaflets and booklets
Epidemiological and entomological examination of malaria foci
Use of IRS and LLINs
Seminars and workshops for personnel
